# Quality management: reduction of waiting time and efficiency enhancement in an ENT-university outpatients' department

**DOI:** 10.1186/1472-6963-9-21

**Published:** 2009-01-31

**Authors:** Matthias Helbig, Silke Helbig, Heike A Kahla-Witzsch, Angelika May

**Affiliations:** 1 Department of Otolaryngology, Head and Neck Surgery, University Hospital of Frankfurt/Main, Theodor Stern Kai 7, 60590 Frankfurt/Main, Germany; 2 Supervisory Office of Quality Management, University Hospital of Frankfurt/Main, Theodor Stern Kai 7, 60590 Frankfurt/Main, Germany

## Abstract

**Background:**

Public health systems are confronted with constantly rising costs. Furthermore, diagnostic as well as treatment services become more and more specialized. These are the reasons for an interdisciplinary project on the one hand aiming at simplification of planning and scheduling patient appointments, on the other hand at fulfilling all requirements of efficiency and treatment quality.

**Methods:**

As to understanding procedure and problem solving activities, the responsible project group strictly proceeded with four methodical steps: actual state analysis, analysis of causes, correcting measures, and examination of effectiveness. Various methods of quality management, as for instance opinion polls, data collections, and several procedures of problem identification as well as of solution proposals were applied. All activities were realized according to the requirements of the clinic's ISO 9001:2000 certified quality management system. The development of this project is described step by step from planning phase to inauguration into the daily routine of the clinic and subsequent control of effectiveness.

**Results:**

Five significant problem fields could be identified. After an analysis of causes the major remedial measures were: installation of a patient telephone hotline, standardization of appointment arrangements for all patients, modification of the appointments book considering the reason for coming in planning defined working periods for certain symptoms and treatments, improvement of telephonic counselling, and transition to flexible time planning by daily updates of the appointments book. After implementation of these changes into the clinic's routine success could be demonstrated by significantly reduced waiting times and resulting increased patient satisfaction.

**Conclusion:**

Systematic scrutiny of the existing organizational structures of the outpatients' department of our clinic by means of actual state analysis and analysis of causes revealed the necessity of improvement. According to rules of quality management correcting measures and subsequent examination of effectiveness were performed. These changes resulted in higher satisfaction of patients, referring colleagues and clinic staff the like. Additionally the clinic is able to cope with an increasing demand for appointments in outpatients' departments, and the clinic's human resources are employed more effectively.

## Background

Public health systems are confronted with constantly rising costs and diagnostic as well as treatment services become more and more specialized. On the other hand resources (staff and finances) are becoming tighter, whereas more and more patients seek treatment in the clinic. Consequently, purposeful planning and demand-oriented scheduling of patient appointments in the outpatients' departments and specialty consulting hours gain more and more importance. Therefore, to keep treatment efficiency and quality warranted [1-3], highly specialized treatments as well as those for very rare illnesses and diseases with complicated healing process have to be integrated into an overall plan [4,5]. To cope with this changing general setup of the clinic's daily routine organizational and structural changes have to be realized and the introduction of new regulatory mechanisms is indispensable. Patient complaints, results from a previous patient opinion poll as well as from an inquiry about waiting times, and verbal communications of referring doctors as well as our clinic's personnel made clear that the organization of our outpatient's department showed deficits, especially in consultation scheduling.

Prerequisite of the clinic management was, that according to the requirements of the ISO 9001: 2000 certified quality management system of our clinic [6-10] the appointment scheduling of the outpatients' department should be improved with respect to efficiency and service quality. So, the duty of the established interdisciplinary task force was the systematic analysis of deficits in our appointment scheduling practice by means of quality management measures. Inefficient procedures had to be analyzed and improvements to be developed. Aim was to simplify scheduling of patient appointments, to assign patients more purposefully to speciality consulting hours, and to allow for more consultation time for patients and referring doctors the like. Then these remedial measures had to be integrated into daily clinical routine. Finally the practicability of these procedures had to be investigated.

This article describes and discusses how quality management instruments can be applied to scrutinize and improve existing work flow procedures.

## Methods

The whole project was realized by a task force consisting of two doctors, two members of the nursing services and one administrative assistant. Periodical one hour team conferences were held every two weeks. The time frame of the innovation project (except preliminary patient polls and re-evaluation) was scheduled for a period of six months. The activities were organized into four methodical phases:

• actual state analysis

• analysis of causes

• correcting measures

• examination of effectiveness

### Actual state analysis

Interpretation of a preliminary patient opinion poll: This poll was carried out two years before by a member of the quality management executive board of the clinic and focussed on patient satisfaction with the hospital treatment. The patients were asked to fill out a questionnaire and put it anonymously into a ballot box. Three questions concerning the outpatients' department from the complex questionnaire were interpreted here: 1. subjective perception of waiting time, 2. overall impression and 3. wish for future appointments in our clinic. The interrogation period was four weeks, 136 forms could be analyzed.

Analysis of an outpatients' poll concerning waiting time: This opinion poll was conducted over three weeks and one year previously by an intern together with a member of the administrative team. Patients with scheduled appointments (no emergencies) were asked about their waiting time at the receptionist's, the time passed by between registration and treatment, and the duration of their treatment. The answers were written down and this document was attached to the patient's file. 222 out of 285 answers could be included in the evaluation.

Beginning with the actual problem solving task the following analysis of strengths and weaknesses was carried out within a period of two months.

Verbal interviews: To update the results of these previous opinion polls, 40 patients and 10 referring doctors were asked at the beginning of this project to get an overall impression of their satisfaction with appointment scheduling and organization of the outpatients' department's working hours. In parallel, 15 staff members were interrogated as to their satisfaction with work flow and organization of the outpatients' department. Referring doctors and personnel were interviewed by an intern whereas the patient questionings were conducted by a member of the nursing service. The results of these interviews were taken down, assigned to the different topics and thus were basis for the analysis of causes.

Investigation of mean work load: The time involved for treatment of the ten most common appointment reasons was measured with a stopwatch. Medical and non-medical activities were registered separately. These results were basis for new appointment scheduling rules.

### Analysis of causes

Mind mapping and cause and effect diagrams were used to identify essential problem fields and to correlate them to their respective causes. These results served as basis for the elaboration of correcting and improving changes. The analysis of causes was done in several consecutive meetings of the task force during the two months after interpretation of the actual state analysis.

### Correcting measures

Formulation of correcting actions was then done by the task force in four meetings of the following two months.

Force-field analysis and failure mode and effect analysis were applied to find adequate problem solving proposals and to identify potential weak points of such correcting measures.

Implementation of remedial measures: After approval of the clinic management the necessary infrastructure was made available. This comprised a separate room for the receptionist as well as separate telephone and fax numbers and a computer. Then a nurse was withdrawn from routine work and prepared for her new task. The complete package of correcting and improving actions was introduced on a particular cutoff date.

Information and announcement: The whole clinic staffs were notified of the changes. The single members of the task force were responsible for the problem-free implementation of the different innovations and appropriate instruction of their own professional staff. Externally the new organizational structure was imparted on the clinic's internet website as well as by verbal and written information to referring doctors. Additionally a press report was launched.

### Examination of effectiveness

#### Control of the electronic appointments book

From the beginning of the correction phase, the modified appointments book was controlled and updated daily by the responsible nurse and an intern. So, rare cases of mistakes or lack of information could be corrected in time.

#### Written patient opinion poll

Six months after implementation the degree of satisfaction with the new procedure was checked by a written patient survey. During a period of two weeks 145 scheduled outpatients (no emergencies) received a questionnaire, which they were asked to fill out until the end of their treatment. 126 papers were returned for interpretation. The questions asked concentrated on availability of telephonic contact, kind of appointment arrangement, and satisfaction with date and consultation arrangement process. These items could be scored by a system of 1 = "very good" through 5 = "unsatisfactory"). Additional questions concerned the subjective perception of waiting time, the overall impression, and the willingness to schedule future appointments. The answers to these latter questions could be directly compared with the results of the preliminary inquiry (136 patients) with the same questions and the same evaluation method. Furthermore, waiting time at the reception desk, between registration and treatment start, and between beginning and end of the medical treatment were evaluated. These results could be compared to the above mentioned waiting time analysis realized one year before with 222 outpatients.

#### Verbal interviews

The same referring colleagues and the same staff members who were interviewed for the actual state analysis were asked again by the same interviewer six months after implementation of the new system. As this was an open questioning about the organization of the outpatients' ward and appointment scheduling only a protocol was written but no statistics were performed.

## Results

The results of the single project phases are described in the following text and subsumed in table 1.

**Table 1:** Phases of project development with results

	**Analysis of strong and weak points (actual situation)**	**Analysis of causalities**	**Correcting measures**	**Control of effectiveness**
1)	Unsatisfactory possibility of telephone contact	Telephone appointment arrangement done additionally by all personnel of outpatients' department	Establishment of a patient hotline; continuous availability; standardization of appointment arrangement for all patients; clear rules for emergencies after opening hours	Patient survey (*time: 6 months after introduction*)
2)	Little consultation activity	No medical education of administrative staff and too little time	Guaranteed and correct consultation by experienced nurse and medical team	Patient opinion poll; analysis of waiting periods (*time: 6 months after introduction*)
3)	Faulty utilization of office hours and speciality consulting hours	Unknown expected workload because no reason was registered	Modification of the appointments book: illness and/or reason for contact is written down	Analysis of waiting periods, analysis of use to capacity of outpatients' department stuff survey (*time: 6 months after introduction*)
4)	Bad plannability of medical and nursing work flow	No relation between treatment time and symptoms	Appointment arrangement dependent on symptoms or contact reason; time units defined for certain symptoms or treatments	Analysis of waiting period (*time: 6 months after introduction*)
5)	Little flexibility of the system	Rigid appointment arrangement system	Transition to flexible time planning by daily updates of appointments book	Analysis of appointments book (unproductive phases) (*daily*)

### Actual state analysis

Analysis of the previous outpatients' poll concerning waiting time: The interpretation of the 222 questionnaires showed the following results: waiting time at receptionist's: 14 min (standard deviation 3.6), waiting time between registration and treatment start: 57 min (standard deviation 31.9), waiting time between beginning and end of medical treatment: 61 min (standard deviation 26.0).

Interpretation of the preliminary patient opinion poll two years ago: The subjective perception of waiting time was evaluated with a score of 3.1, the overall impression was scored 2.1, and 75% of the interviewed persons uttered the wish for future appointments (Scores from 1 = good through 5 = bad).

This opinion poll among patients, referring colleagues and personnel revealed the following problems:

1) Patients as well as referring doctors pointed out deficits of telephone availability for appointments.

2) Patients as well as referring colleagues were dissatisfied with insufficient consultation activities as to medical and formal questions on the phone.

3) Interns pointed out wrong and insufficient use of capacities of the general and the speciality consulting hours.

4) Interns complained of poor treatment planning in the outpatients' department. Appointments were not deliberately planned and not adapted to the necessary treatment steps.

5) Patients, referring colleagues, and medical as well as non-medical staff were dissatisfied by a definite lack of flexibility as to time needs of appointment scheduling.

Inquiry of the mean work load for the ten most common appointment reasons: Different syndromes cause different work loads and time needs for interns and nurses. Whereas some syndromes are very time-consuming for the therapist, others cause more work load for the nursing staff. These different data are shown in table 2.

**Table 2:** Determination of mean work load for medical and nursing treatment for five out of ten most frequent reasons for visit

**Symptoms**	**Necessary activities**	**Involved**	**Duration (minutes)**
First visit with unclear deafness, chronic vertigo, cholesteatoma	Anamnesis and examination	Intern	15
	Audiological examination	MTA	10
	Examination of the estibular nerve	MTA	15
	Discussion of results, presentation to assistant medical director, planning and decision making	Intern	10
	**Total time needed for intern:**		**25**
	**Total time needed for MTA:**		**25**
First visit with impaired nasal respiration, chronic sinusitis	Anamnesis and examination	Intern	15
	Rhinomanometry and Prick test, taking of blood samples	Nurse	45
	Discussion of results, presentation to assistant medical director, planning and decision making	Intern	10
	**Total time needed for intern:**		**25**
	**Total time needed for nurse:**		**45**
First visit with parotid tumour, swollen neck	Anamnesis and examination	Intern	10
	Sonography and fine needle biopsy	Intern	10
	Assistance in fine needle biopsy	Nurse	05
	Discussion of results, presentation to assistant medical director, planning and decision making	Intern	10
	**Total time needed for intern:**		**30**
	**Total time needed for nurse:**		**05**
First visit with tumour of larynx/pharynx	Anamnesis and examination	Intern	10
	Sonography	Intern	10
	taking of blood samples	Nurse	05
	Discussion of results, presentation to assistant medical director, planning and decision making, appointment arrangement for staging investigation	Intern	15
		Intern	20
		Nurse	**20**
	**Total time needed for intern:**		**35**
	**Total time needed for nurse:**		**25**
Follow-up visit for change of dressings or discussion of histology results	Compilation and evaluation of histology findings	Intern	03
	Change of dressings, examination and patient instruction	Intern	07
	Assistance in change of dressings	Nurse	05
	decision making	Intern	05
	**Total time needed for intern:**		**15**
	**Total time needed for nurse:**		**05**

### Analysis of causes

Four major causes for these problems were identified:

1) Telephonic appointment scheduling was usually done additionally at the reception desk partly by administrative assistants or members of the nursing staff in the outpatients' ward.

2) Patient consultation with respect to medical as well as formal questions was unsatisfactory in some cases, caused by inadequate professional knowledge and lack of time.

3) No comments were registered in the schedule planner as to reason and urgency of an appointment. This inadequate information impeded correct scheduling of medical and nursing treatment as the necessary amount of work could not be estimated in advance. So, possibilities to directly assign certain patients to necessary speciality consultations were limited.

4) Unforeseen work overload as well as sudden lack of challenge in all fields could be possible because appointment planning was too stereotyped and not flexible enough.

### Correcting measures

1) A service point with a patient telephone hotline for appointments and consultations was installed that is available for patients and referring doctors. This service was organized as a "central appointment office" and is manned continuously from 8.00 a.m. to 4.30 p.m. on all working days. Apart from these fixed working hours an answering machine gives clear and unequivocal information as to availability and responsibility during regular off-duty hours. Furthermore first advice how to behave in emergency situations is given. Appointments are arranged in a standardized manner by phone (one definite phone number), by fax (one special fax number), per e-mail, or by personal visit.

2) Provision of competent consultations concerning medical or formal matters was organized. It is important whether the receptionist is a medical person or not. So, appointments are now arranged by a very experienced examined nurse. She has been working in our clinic for many years and is very well acquainted with all its structural and formal conditions. Thus competent consultation as to medical and organizational questions is ensured even in the first contact. In addition, she can rely on the assistance of the medical interns and the assistant medical director on duty in case of medical in-depth questions.

3) Modifications of the appointments book were planned to make allowance for structural circumstances and human resources of the clinic (e.g. speciality consulting hours or presence of certain interns). One important modification was that his/her phone number is added to the patient's name. Thus, the patient can be contacted in case of necessary short-time changes or if further questions should arise. Additionally, the reason for coming (first contact or re-consultation, change of dressings, discussion of histology, etc.) and, if applicable, the doctor's name of the last appointment are registered.

4) Appointment planning in dependence of the diagnosis or the consultation reason was optimized. The prospective treatment time is already taken into account when a consultation is planned. This applies especially when it becomes clear during the contact call that this special consultation or treatment will be more time-consuming. On the other hand, even short term consultations can be arranged. The different doctor-patient contacts were planned by means of so-called "time windows" in the appointments book. The duration and amount of work of the ten most frequent reasons for coming were defined with respect to medical and nursing activities (table 2). For all other symptoms a time span of 20 minutes is calculated for medical treatment and of 15 minutes for nursing activities. Patients asking for a follow-up appointment are assigned to the doctor who knows this patient from previous consultations. Patients with special syndromes are assigned to specialized interns. This allows for a purposeful examination; unnecessary delays from studying the medical report as well as further inquiry can be avoided. Thus, patients get their necessary appointments, meeting the needs of the symptoms and the resources of the clinic alike.

5) Appointment planning was made more flexible to make the patients' wishes meet the structural and organizational prerequisites of the clinic. Additionally, short-term cancellation times can be filled with other patients by telephonic contact.

### Efficiency control

During the initial phase of the project (first month) efficiency and interface coordination were checked daily by the members of the task force. The strict control of the new behaviour showed good integration into the clinic's routine and organization, no remarkable aberration from the guidelines was to be seen. All requirements were fulfilled satisfactorily.

The consequent daily update and control of the electronic appointments book makes it possible to react directly to unforeseen events as e. g. cancellation of appointments, additional short-term consultation wishes or emergency cases. The controls showed that such last minute changes occur daily.

Written patient interviews six months later revealed high grades of satisfaction with the new organization form. The scores are for telephonic availability 1.7, for kind of appointment arrangement 1.7, for satisfaction with date 1.8, for arrangement process 2.0 (table 3). The subjective perception of waiting time was scored 2.5, the overall impression 2.1 and 95% of the patients declared to contact again for necessary new appointments. Compared to our opinion poll from two years ago all these scores improved noticeably (table 4). Furthermore, the average waiting periods of seven minutes (standard deviation 3.1) at the receptionist's improved by 50% in comparison with the opinion poll from one year ago. The period between registration and treatment start improved from 57 to 45 minutes (standard deviation 13.2, mean time gain of 21%). The time between treatment start and end was 36 minutes (standard deviation 14.3). This is a mean time gain of 25 minutes (= 41%) compared to the result from the previous year (table 5).

**Table 3:** Results of patient survey regarding satisfaction and acceptance of the newly installed patient hotline.

Questions concerning:	Overall assessment Survey six months after implementation (n = 126 patients)
Availability of telephonic contact	1.7
Kind of appointment arrangement	1.7
Satisfaction with date	1.8
Satisfaction with appointment arrangement process	2.0

Subjective degrees (from 1 = very good to 5 = unsatisfactory) to be assigned by the patients.

**Table 4:** Comparison of results of patient surveys.

Questions concerning:	Overall assessment survey 2 years **before **implementation (n = 136 patients)	Overall assessment survey 6 months **after **implementation (n = 126 patients)
subjective perception of waiting time	3.1	2.5
the overall impression	2.6	2.1
wish for future appointments	yes: 75%	yes: 95%

Subjective degrees (from 1 = very good to 5 = unsatisfactory) to be assigned by the patients.

**Table 5:** Comparison between two patient surveys regarding waiting period in the ENT outpatients' department.

	Survey 1 year **before **establishment of the measures (n = 222 patients)	Survey 6 months **after **establishment of the measures (n = 126 patients)		
Waiting period	Waiting period (min)	Waiting period (min)	mean time gain (min)	mean time gain (%)
at reception desk	14 *(SD: 3.6)*	7 *(SD: 3.1)*	7	50
between registration and treatment start	57 *(SD: 31.9)*	45 *(SD:13.2)*	12	21
between beginning and end of medical treatment	61 *(SD: 26.0)*	36 *(SD: 14.3)*	25	41

(Standard deviation = SD)

An internal verbal questioning of the intern team, the administrative team and the nursing team of the outpatients' ward conducted six months after implementation of the system revealed a high grade of satisfaction with the working conditions in the ward.

## Discussion

The general situation of public health systems forces clinical institutions to take regulatory measures as to their organization and structure. Only by such changes will it be possible to maintain the claim of efficient and high-quality treatment [11]. Such efforts including their problems are described here.

This project was realized within strict time limits. Furthermore, the personal, financial and organizational set-up of the clinic had to be taken into account. Nevertheless, an interdisciplinary task force could pinpoint structural deficits of the outpatients' department of the ENT clinic of the Johann-Wolfgang-Goethe University, Frankfurt, Germany. The whole procedure was performed by means of established quality management methods according to the requirements of our ISO 9001:2000 certified QM management system. Though there are more sophisticated methods for actual state analysis, open interviews proved valuable enough to obtain concrete data. According to these answers inefficient procedures in the actual system were analysed and improvement proposals were developed. Creative mind mapping and effect diagrams as tools of identification of problems quickly lead to several important causes for the actual inefficiencies. Especially force-field analysis and failure mode and effect analysis are apt instruments to find adequate solutions and detect their possible weak points. With respect to the limited resources of the clinic it seemed wise to concentrate on a few elementary changes. The following essential changes were planned: 1) implementation of a constantly manned patient telephone hotline with standardized appointment rules, 2) warranted competent telephonic advice by an experienced nurse, 3) modification of the appointments book 4) appointment scheduling depending on the reported illness and the presumable time needed, 5) flexibility of scheduling.

Central point of the restructuring was the implementation of a telephone patient hotline. This hotline should be installed in a separate room, accessible for patients and ideally as near as possible to the outpatient's department. In our case, this room could be provided; telephone and fax connexions as well as internet access were already installed. Competent advice could be provided by the experienced nurse who has already been in our clinic for a long time. So, she was very well acquainted with all work flows of the separate wards. Fortunately, she had some foreign language knowledge, too. So, an elaborate instruction was not necessary in our case, which otherwise is indispensable. For medical questions members of the intern team were at her disposal. Furthermore, a locum was trained. This made continuous high quality service of this new department possible. A very important advantage of this procedure is that even before the actual consultation of an intern several questions can be cleared in advance and patients can be asked to bring previous diagnosis and medical records with them to avoid unnecessary re-consultations [12]. Another essential change is the restructuring of the appointments book. We continued to use the familiar electronic appointments program. Only some new features were added, so that some additional information must be noted. The whole staff mastered the new version without explicit additional training. Thorough measurements of time consumption of several most common consultation reasons made time planning considerably more reliable [13]. From these measurements we defined several illness-related time windows. So, knowing the precise problems of the patient the receptionist is able to add the presumable time needs for the scheduled appointment. Registration of wishes and/or complaints enables the receptionist to assign certain patients directly to specialized interns, thus additionally saving general consultation time in the ward and, on the other hand, reducing waiting time for the following patients. This adequate and problem-oriented planning respecting wishes of patients and referring doctors on the one hand and orienting on organizational structures as well as limited personal resources of the clinic on the other hand lead to significant improvements of patient satisfaction [14,15]. Outpatient and even inpatient treatment could be coordinated more effectively by optimizing and economizing work flows in the outpatients' ward [16,17]. An additional advantage is the chance to react flexibly to unforeseen irregularities and their adaptation to the resources of the clinic. The receptionist nurse has the possibility to arrange last minute appointments with patients from a waiting list in cases of short-term cancellations or sudden illness of staff members. Thus, unproductive phases often can be avoided.

The task of this project and the issue of this study result from dissatisfaction of patients, interns, and nursing staff with the then actual work flow. In the end, when we organized another opinion poll, we consequently used the same questions as before. So, the answers of both interviews were comparable and therefore are a reliable measurement of the effectiveness of our organizational changes.

Primary purpose of these actions was not to increase the number of treated patients but to cope with the changed amount of outpatients against the background of restricted personal and financial resources of the clinic. Nevertheless the average number of treated patients per intern rose after restructuring of the appointments book thus increasing the service capacity of the clinic. As, however, exact figures from the past were not available, we abstain from figures here, as well. Though this fact was stated by all staff members the main focus of our examination of effectiveness was on satisfaction of all persons involved.

The newly defined tasks of the receptionist nurse are time-consuming enough to keep her the whole day busy. As her work makes life easier for the outpatients' department staff, work flow there is more pertinent. The negative aspect of this restructuring is that the manpower of an experienced nurse is withdrawn from patient treatment. Each clinic has to decide if such a solution is affordable in their special situation. On the other hand the good acceptance of a permanent and competent partner on the part of patients and referring doctors alike is reflected by the results of our following opinion poll. Last but not least loss of time at the receptionist's, between registration and treatment start, and between beginning and end of the medical treatment was considerably cut down and appreciated by the interviewed persons.

## Conclusion

The step-by-step description of the project phases is to illustrate our systematic approach in coping with the problem. The concrete procedure employed methods of quality management. Our changes resulted in increased efficiency of work flows and high grades of satisfaction of patients, referring colleagues and personnel. So, this presentation of planning and implementing concrete correcting actions as well as subsequent control of their efficiency may be helpful to colleagues confronted with similar problems in their clinic. Purpose is to give practical improvement hints how to optimize appointment scheduling and patient consulting in an outpatients' department. The addressed problems arising with such a task and the positive outcome of our efforts allow taking these practical suggestions as blueprint for similar projects.

## Competing interests

The authors declare that they have no competing interests.

## Authors' contributions

MH conceptualised the study, conducted data analysis, wrote and revised the manuscript. SH performed the "actual state analysis", collected data and conducted the data analysis. HAKW supervised the study. AM participated in the design of the study, collected and analysed data and performed the literature review. All authors read and approved the final manuscript.

## Pre-publication history

The pre-publication history for this paper can be accessed here:


